# Inflammasome-Mediated Inhibition of *Listeria monocytogenes*-Stimulated Immunity Is Independent of Myelomonocytic Function

**DOI:** 10.1371/journal.pone.0083191

**Published:** 2013-12-09

**Authors:** Cassandra R. Williams, Michael L. Dustin, John-Demian Sauer

**Affiliations:** 1 Molecular Pathogenesis Program, The Helen L. and Martin S. Kimmel Center for Biology and Medicine at Skirball Institute of Biomolecular Medicine, New York, New York, United States of America; 2 Department of Medical Microbiology and Immunology, University of Wisconsin-Madison, Madison, Wisconsin, United States of America; University of Illinois at Chicago College of Medicine, United States of America

## Abstract

Activation of the Nlrc4 inflammasome results in the secretion of IL-1β and IL-18 through caspase-1 and induction of pyroptosis. *L. monocytogenes* engineered to activate Nlrc4 by expression of *Legionella pneumophilia* flagellin (*L. monocytogenes L*.p.*FlaA*) are less immunogenic for CD8^+^ T cell responses than wt *L. monocytogenes*. It is also known that IL-1β orchestrates recruitment of myelomonocytic cells (MMC), which have been shown to interfere with T cell-dendritic cells (DC) interactions in splenic white pulp (WP), limiting T cell priming and protective immunity. We have further analyzed the role of MMCs in the immunogenicity of *L. monocytogenes L*.p.*FlaA*. We confirmed that MMCs infiltrate the WP between 24–48 hours in response to wt *L. monocytogenes* infection and that depletion of MMCs enhances CD8^+^ T cell priming and protective memory. *L. monocytogenes* L.p.FlaA elicited accelerated recruitment of MMCs into the WP. While MMCs contribute to control of *L. monocytogenes L*.p.*FlaA*, MMC depletion did not increase immunogenicity of L.p.FlaA expressing strains. There was a significant decrease in *L. monocytogenes* L.p.FlaA in CD8α^+^ DCs independent of MMCs. These findings suggest that limiting inflammasome activation is important for bacterial accumulation in CD8α^+^ DCs, which are known to be critical for T cell response to *L. monocytogenes*.

## Introduction

Pattern recognition receptors (PPR) are germ-line encoded components of innate immunity that bind various conserved microbial components such as lipopolysaccharide (LPS), lipoproteins, lipoteichoic acid (LTA), peptidoglycan and flagellin, collectively referred to as pathogen-associated molecular patterns (PAMPs) [1]. PRRs include Toll-like receptors (TLR) that survey PAMPs at the cell membrane and in vacuoles and Nod-like-receptors (NLR) that detect PAMPs in the cytosol [2,3]. A subset of NLR family proteins has been shown to assemble into an inflammasome complex leading to the activation of caspase-1 [4]. Caspase-1 activation results in the activation and secretion of IL-1β and IL-18 as well as the induction of caspase-1-dependent cell death, also known as pyroptosis [5]. One of the most well characterized inflammasomes, the Nlrc4 inflammasome (also known as Ipaf), activates caspase-1 in response to contamination of the cytosol with either bacterial flagellin or type III secretion inner rod proteins through receptors NAIP5 and NAIP2, respectively [6-10]. Release of cytokines, chemokines and other endogenous inflammatory mediators leads to recruitment of innate immune cells such as neutrophils and monocytes that contribute to containing infection [11]. 

*Listeria monocytogenes* is a Gram-positive, facultative intracellular pathogen that has been used extensively to study cell-mediated immunity [12-17]. As a requirement for efficient cell-to-cell movement, *L. monocytogenes* uses virulence factor ActA (*actA* gene) to hijack host actin polymerization machinery propelling itself into the host cell plasma membrane for subsequent engulfment by a neighboring cell [18]. ActA deficient strains are 1000 fold less virulent than ActA sufficient strains, but still induce robust CD8^+^ mediated immunity and memory [19]. Internalin B (*inlB* gene) is a *L. monocytogenes* virulence factor that binds c-met, the natural receptor for hepatocyte growth factor (HGF), promoting invasion of non-phagocytic cell types and liver colonization [20,21]. *L. monocytogenes* vaccine strains often have both *inlB* and *actA* genes deleted (*ΔactA/ΔinlB*) to reduce toxicity while retaining stimulation of adaptive cellular immunity[19]. 

*L. monocytogenes* is a stealthy pathogen in that it enters the cytoplasm with minimal activation of the Nlrc4 inflammasome, in part by down-regulating its flagellin genes upon entry into the host [22,23]. Previously, we engineered a strain of *L. monocytogenes* to specifically activate the Nlrc4 inflammasome by linking secretion of *Legionella pneumophila* flagellin to the *actA* regulatory elements so that flagellin is expressed upon entry into the cytoplasm [23]. This strain (*L. monocytogenes L*.p.*FlaA*) was 1000-fold less virulent than wt strains during acute infection and was defective at inducing long-term cell-mediated immunity [23]. The mechanisms by which the inflammasome attenuates acute infection or leads to a decrease in long-term cell-mediated immunity are not understood. In infections such as *Staphylococcus aureus* where neutrophil recruitment is required for the elimination of the pathogen [24,25], inflammasome-mediated production of IL-1β has been shown to promote neutrophil recruitment at the site of infection [11], thus implicating these cells in the virulence *L. monocytogenes* L.p.FlaA defect. 

Current literature provides conflicting data on the individual roles of myelomonocytic cells (MMC), consisting of neutrophils and monocytes, in defending against infections with *L. monocytogenes* as well as their roles in establishing *L. monocytogenes*-specific CD8^+^ cytotoxic T cells (CTLs) [26]. Reports have shown that neutrophils are responsible for controlling *L. monocytogenes* infection through engulfment of extracellular bacteria, followed by the generation of reactive oxygen and nitrogen intermediates [27]. Neutrophils have also been shown to migrate into the draining lymph nodes shortly after immunization and compete with antigen-presenting cells for antigen, decreasing antigen inside DCs and abrogating DC-T cell interactions [28,29]. In contrast, Shi et al. found through selective depletion that neutrophils were dispensable and that Ly6C^hi^ inflammatory monocytes were essential for bacterial control during innate responses to *L. monocytogenes*. The selective depletion of inflammatory monocytes also resulted in the impairment of IFN-γ expression by CD4^+^ T cells [30]. Miao et. al. suggested that the attenuation of *Salmonella typhimurium* engineered to activate the inflammasome was independent of IL-1β and IL-18 and that caspase-1-induced pyroptotic cell death released bacteria from macrophages and exposed the bacteria to uptake in neutrophils and subsequent killing by reactive oxygen species [9]. 

Previously, we found that antibody-mediated depletion of neutrophils did not rescue the virulence of *L. monocytogenes* L.p.FlaA [23], therefore, we wanted to examine the possible role of both neutrophils and Ly6C^hi^ monocytes in bacterial containment and CD8^+^ T cell generation during infections with *L. monocytogenes L*.p.*FlaA*. As such, we measured changes in MMC migration into splenic WP after inflammasome activation and established the effect of early neutrophil and monocyte depletion on bacterial clearance and CD8^+^ T cell expansion. We found that inflammasome activation reduces the bacterial load in CD8α^+^ DC, which correlates with the attenuated CD8^+^ T cell response and recall response. Thus, low inflammasome activation is an important feature of *L. monocytogenes* immunogenicity that can be dissociated from overall bacterial burden based on the differential residence of bacteria in DC subsets.

## Results

### Myelomonocytic depletion increases CTL generation and protective immunity

**Figure 1:**
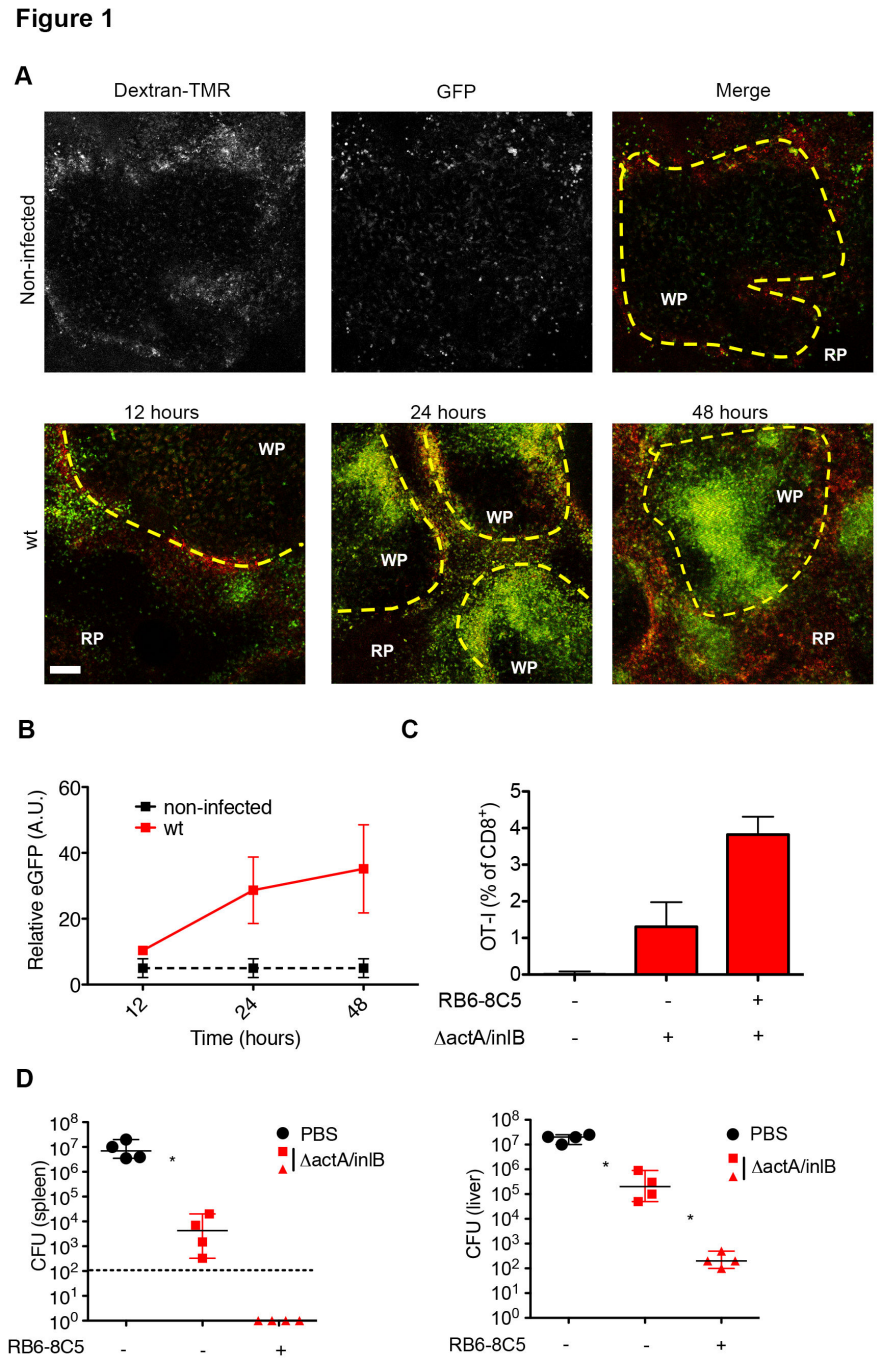
Increased CD8^+^ T cell generation and protective immunity after MMC depletion. (A) 8-10 week old LysM^+/eGFP^ mice were infected intravenously with 2.5x10^4^ wt *L. monocytogenes* for 12, 24, and 48 hours. At the indicated time spleens were excised, sectioned, and image using multi-photon microscopy. Multi-photon images of LysM^+/eGFP^ mice at 12, 24, and 48 hours after infection. Top panel (non-infected) –grayscale images. Dextran-TMR-red, LysM^+/eGFP^- green, yellow dotted line-marginal zone, WP-white pulp, RP-red pulp (B) Relative GFP intensity inside individual WP nodule (A.U.). Data representative of 3 independent experiments. (C) Isolated naïve OT-I cells were transferred into 8-10 week old B6.SJL mice 24 hours prior to 250µg RB6-8C5 mAb. 5 hours after mAb treatment mice were infected i.v. with 1x10^4^ *ΔactA/InlB* *L. monocytogenes* expressing ova peptide. OT-I percentages as analyzed by FACs from blood day 7 post-infection. Bar graph (median plus range). Data representative of 3 independent experiments. (D) 8-10 week old C57BL/6 mice were treated with 250µg RB6-8C5 mAb and then immunized 5 hours later with 1x10^3^ ΔactA/InlB. 30 days post-immunization, mice were infected with 2x10^5^ wt *L. monocytogenes* for 3 days. Bacterial CFUs in liver and spleen day 3 post-infection. Dotted line – limit of detection. Data are representative of four independent experiments. *P < 0.05 by Mann-Whitney test.

To understand the kinetics of MMC migration following *L.monocytogenes* infection, we first examined the infiltration of MMCs into splenic WP at various times post-infection. Splenic white pulp is lymphocyte-rich and a major site for T cell activation [31,32]. Using an adaptation of a previously described *ex vivo* imaging technique that allows for the visualization of splenic WP [33,34], we imaged sectioned spleens from LysM^+/eGFP^ (eGFP labeled MMCs) mice infected with 2.5 x 10^4^ *L. monocytogenes* for up to 48 hours post infection. We found that upon infection with *L. monocytogenes*, MMCs steadily infiltrated into splenic WP, creating a focus of MMC infiltration around the central arteriole (Figure 1A). The infiltration of MMCs increased significantly over time as indicated by measurement of GFP intensity (Figure 1B). Thus, the LysM^+/eGFP^ mice report MMC infiltration during *L. monocytogenes* infection with a time course consistent with prior studies [16].

MMCs are reported not only to have a role in bacterial containment, but also to be involved in the generation of cell-mediated immunity [29,30]. In order to examine this role, we first looked at the ability of MMCs to alter priming of CD8^+^ T cells. To do this, we adoptively transferred 5x10^4^ naïve, OVA specific, OT-I CD8^+^ T cells from C57BL/6 mice into B6.SJL mice, bearing a distinct CD45 allotype. 24 hours after transfer, mice were treated with PBS (non-infected) or 1.0x10^3^ *L. monocytogenes ΔactA/ΔinlB*-OVA with and without RB6-8C5 mAb treatment. High dosages of RB6-8C5 mAb (250μg) were used to deplete both Ly6C^hi^ monocytes and neutrophils and were injected i.p. 5 hours prior to infection [16,30]. OT-I T cell expansion was monitored by determining the percentage of OT-I in the blood by flow cytometry 7 days post-infection. The OT-I T cells were undetectable (0.03±0.05%) in blood of non-infected animals, and MMC depletion increased OT-I T cell expansion from 1.5±0.43% to 3.7±0.74% (Figure 1C). Thus, our results are consistent with recent work in vaccine models that MMC attenuate CD8^+^ T cell priming [29].

To establish the effect of MMC depletion on protective immunity, we treated mice with RB6-8C5 antibody 5 hours prior to i.v. injection with PBS or 1x10^3^ *L. monocytogenes ΔactA/ΔinlB*. Mice were re-infected with 2x10^5^ (2xLD_50_) *L. monocytogenes* 30 days post immunization and 72 hours later bacterial colony counts were determined in the liver and spleen. Low titer *L. monocytogenes ΔactA/ΔinlB* infection provided 10^2^-10^3^ fold protection in the liver and spleen (Figure 1D). MMC depletion, at the time of immunization, provided an addition 10^3^-10^4^ fold reduction in CFU in the spleen and liver following challenges (Figure 1D). Our results support established data and further implicate the MMC population in the modulation of cell-mediated immunity through its functional role in bacterial clearance. The combined influence of MMCs over immunogenicity led us to investigate their role in the attenuation of *L. monocytogenes* L.p.FlaA and diminished cell-mediated response seen after inflammasome activation.

### Inflammasome activation leads to reduced MMC accumulation in splenic WP

**Figure 2:**
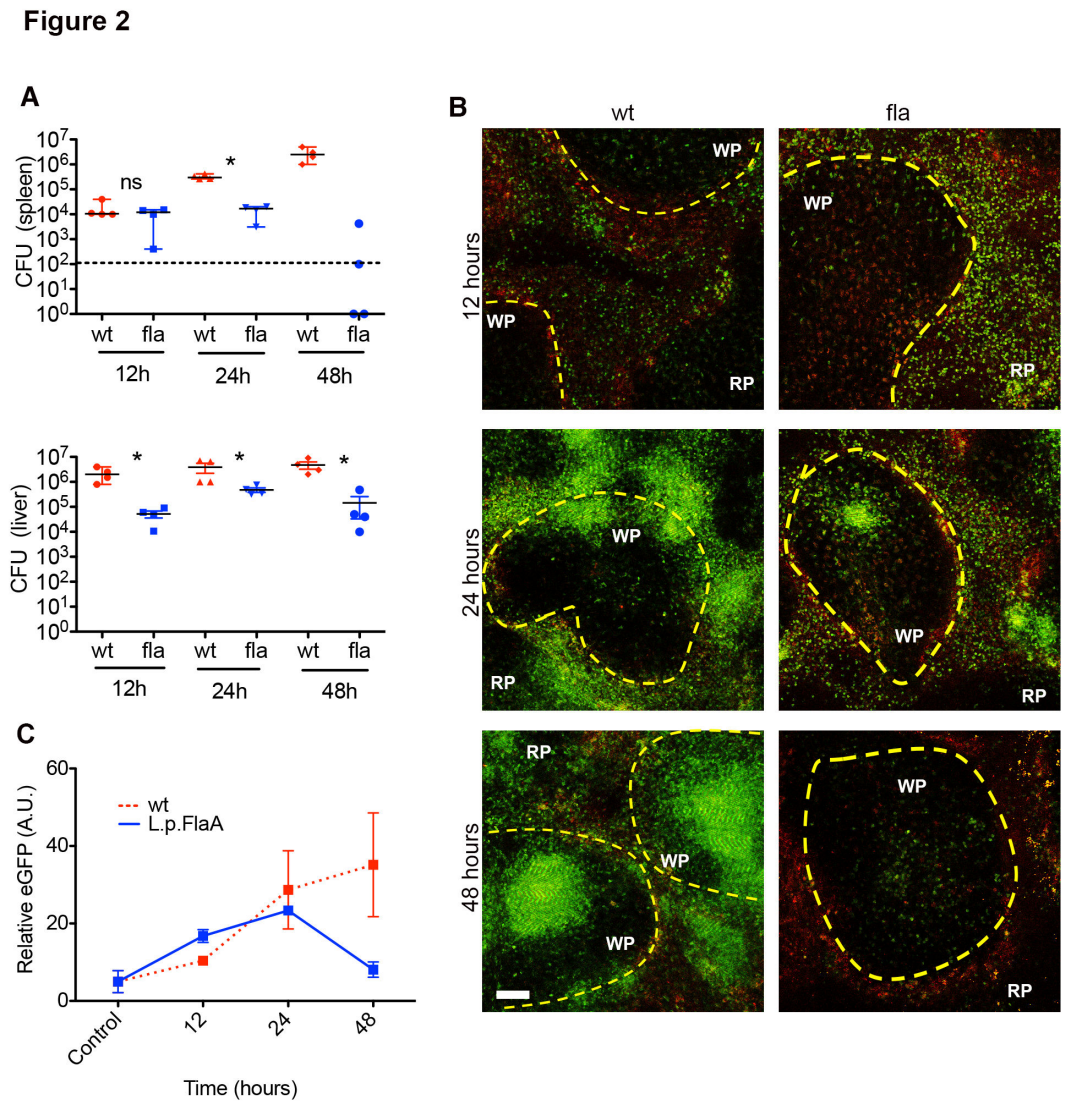
Reduced MMC accumulation after Inflammasome activation. (A) 8-10 week old C57BL/6 mice were infected intravenously with 2.5x10^4^ wt or 1x10^5^ L. *monocytogenes* L.p.FlaA for 12, 24, and 48 hours. Bacterial CFU from spleen and liver were collected at the indicated time points. Dotted line – limit of detection. The median of four replicates from 4 independent experiments. *P < 0.05 by Mann-Whitney test. (B) LysM^+/eGFP^ mice were infected intravenously with 2.5x10^4^ wt or 1x10^5^ L. *monocytogenes* L.p.FlaA for 12, 24, and 48 hours. At the indicated time spleens were excised, sectioned, and image using multi-photon microscopy. Multi-photon images of LysM^+/eGFP^ mice at 12, 24, and 48 hours after infection. LysM^+/eGFP^- green, yellow dotted line-marginal zone, WP-white pulp, RP-red pulp (C) Relative GFP intensity inside individual WP nodule (A.U.). Data representative of 3 independent experiments.

To determine if inflammasome activation alters the recruitment of MMCs to the spleen following immunization, LysM^+/eGFP^ mice were infected with 2.5x10^4^ *L. monocytogenes* (wt) or 1x10^5^ *L. monocytogenes* L.p.FlaA (fla) for up to 48 hours. Previous results established that the *L. monocytogenes* L.p.FlaA strain was highly attenuated [23]. Therefore, to normalize the infection to that of the wt we infected mice with a 4-fold higher inoculum of *L. monocytogenes L*.p.*FlaA*. The increased inoculum of *L. monocytogenes* L.p.FlaA resulted in similar splenic bacterial burden of the parental strain at 12 hours (Figure 2A). After infection, spleens were excised, sectioned, and imaged using the adopted *ex vivo* imaging protocol. MMCs infiltrated the WP earlier, but at lower numbers and for a shorter duration in mice infected with *L. monocytogenes* L.p.FlaA when compared to mice infected with *L. monocytogenes* as determined by measuring the intensity of eGFP fluorescence in the WP (Figure 2B,C). MMC infiltration peaked by 24 hours after *L. monocytogenes* L.p.FlaA infection with MMCs typically penetrating to the deep T cells zone around the pariarteriolar lymphatic sheath, which was not seen with *L. monocytogenes* infection (Figure 2B,C). By 48 hours, MMC accumulation in mice infected with *L. monocytogenes* L.p.FlaA was dramatically reduced while MMCs in the *L. monocytogenes* infection continued to climb (Figure 2B,C). This was correlated with a 5-fold reduction in the splenic bacterial burden by 48 hours post-infection (Figure 2A). This is in contrast to the continued increase in MMC accumulation and bacterial burden in the liver and spleen in *L. monocytogenes*-infected mice (Figure 2A-C). Taken together, these data are consistent with previous reports that *L. monocytogenes* L.p.FlaA are attenuated [23] and suggest that premature infiltration of MMCs into splenic WP could contribute to early clearance of *L. monocytogenes L*.p.*FlaA*.

### MMC depletion partially restores the virulence of *L. monocytogenes* L.p.FlaA

**Figure 3:**
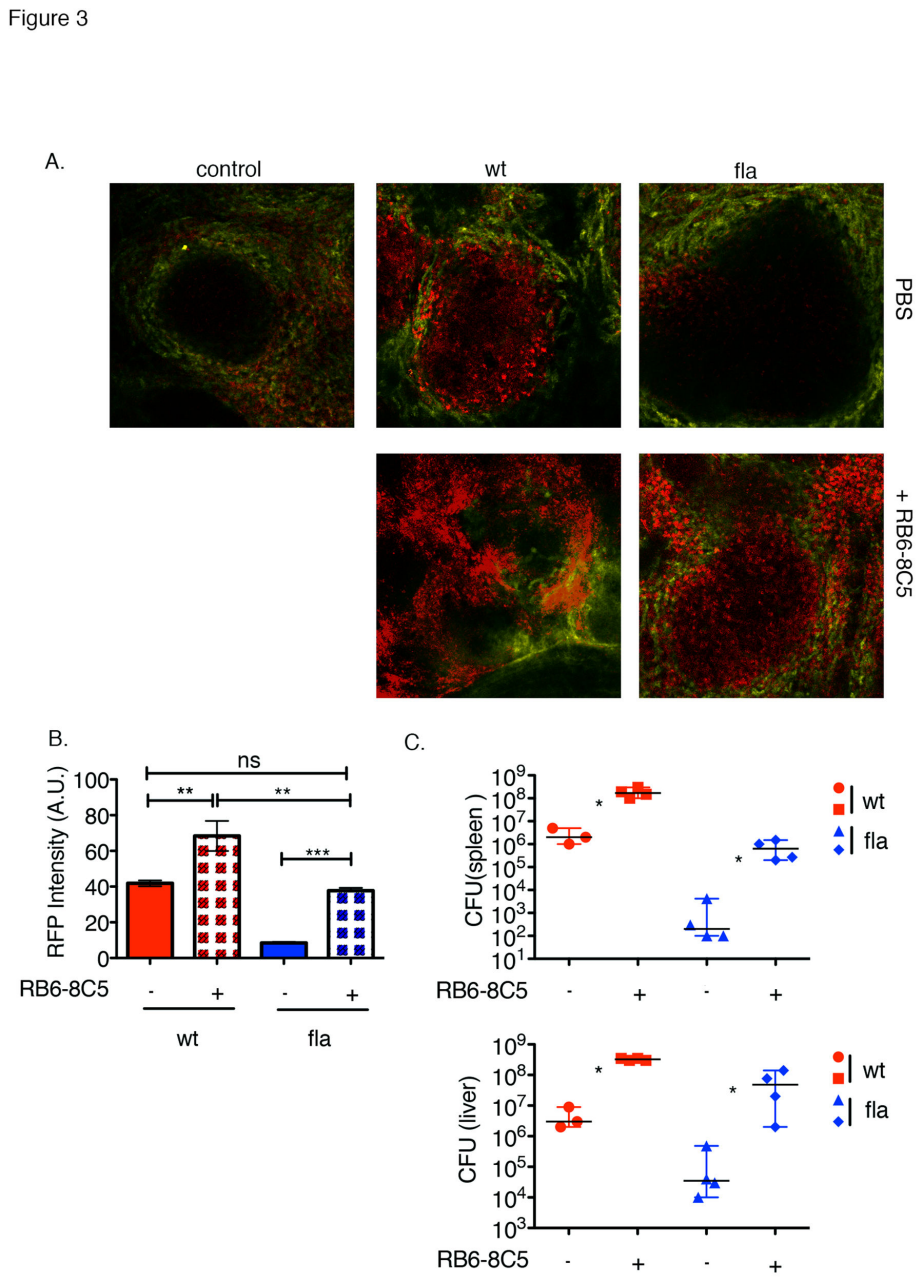
Partial restoration of *L*. *monocytogenes* L.p.FlaA virulence after MMC depletion. 8-10 week old CD11c-YFP transgenic x LysM^+/eGFP^ mice were treated with 250µg RB6-8C5 mAb and then infected i.v. with 2.5x10^4^ wt or 1x10^5^ L. *monocytogenes* L.p.FlaA both expressing RFP for up to 48 hours. After infection spleens were excised, sectioned, and image using multi-photon microscopy. (A) Multi-photon images of CD11c-YFP transgenic x LysM^+/eGFP^ mice at 48 hours after infection. *L. monocytogenes*-RFP, MZ-705 quantum dots-yellow. (B) Relative RFP intensity inside individual WP nodule (A.U.). Data representative of 3 independent experiments. (C) Bacterial CFU from liver and spleen at the indicated time points after RB6-8C5 antibody treatment. The median of at least three replicates from 3 independent experiments. *P < 0.05 by Mann-Whitney test.

MMCs have previously been shown to have an important role in bacterial containment [9,30,35]. Our observation that MMCs prematurely infiltrate the T cell zone during infection with inflammasome-activating *L. monocytogenes* L.p.FlaA led us to ask if MMC depletion would restore the virulence of *L. monocytogenes* L.p.FlaA. Results from bacterial competition indexes had shown that neutrophil depletion using low doses of RB6-8C5 mAb provided no restoration of virulence in mice infected with flagellin expressing *L. monocytogenes* compared to wt *L.monocytogenes* [23]. Low doses of RB6-8C5 are selective for neutrophils [16,36]. Increasing the dose of RB6-8C5 antibody depletes inflammatory monocytes in addition to neutrophils [16,37]. To deplete early infiltrating LysM^+^ cells, we treated CD11c-YFP transgenic x LysM^+/eGFP^ mice with a high dosage (250 μg) of RB6-8C5 mAb previously demonstrated to delete neutrophils and inflammatory monocytes [16]. Mice were then infected with either 2.5x10^4^ *L. monocytogenes* or 1x10^5^ *L. monocytogenes L*.p.*FlaA*, both expressing RFP under control of the *actA* promoter. At 48 hours after infection, spleens were excised, sectioned and imaged using multi-photon microscopy. RFP intensity and colony counting analysis were used to determine bacterial burden inside the white pulp and in the whole spleen and liver, respectively. Red autofluorescence is relatively low in the WP, which facilitated imaging of the signal from the RFP^+^ bacteria (Figure 3A and Figure S1). MMC depletion increased red fluorescence in the WP in both the *L. monocytogenes* and *L. monocytogenes* L.p.FlaA infections (Figure 3A,B). Colony counting from the entire spleen and liver suggested a similar pattern with 10^2^-fold and 10^3^-fold increases in CFU for the *L. monocytogenes* and *L. monocytogenes* L.p.FlaA strains, respectively (Figure 3C). Both the direct imaging and colony counting methods concur that MMC depletion increases *L. monocytogenes* L.p.FlaA burden at 48 hours in the WP and whole spleen, respectively. This increase is similar to that of wt *L. monocytogenes*-infected mice and led us to ask if MMC depletion restores T cell responses. 

### Inflammasome activation inhibits cell-mediated immunity independent of MMC depletion

**Figure 4:**
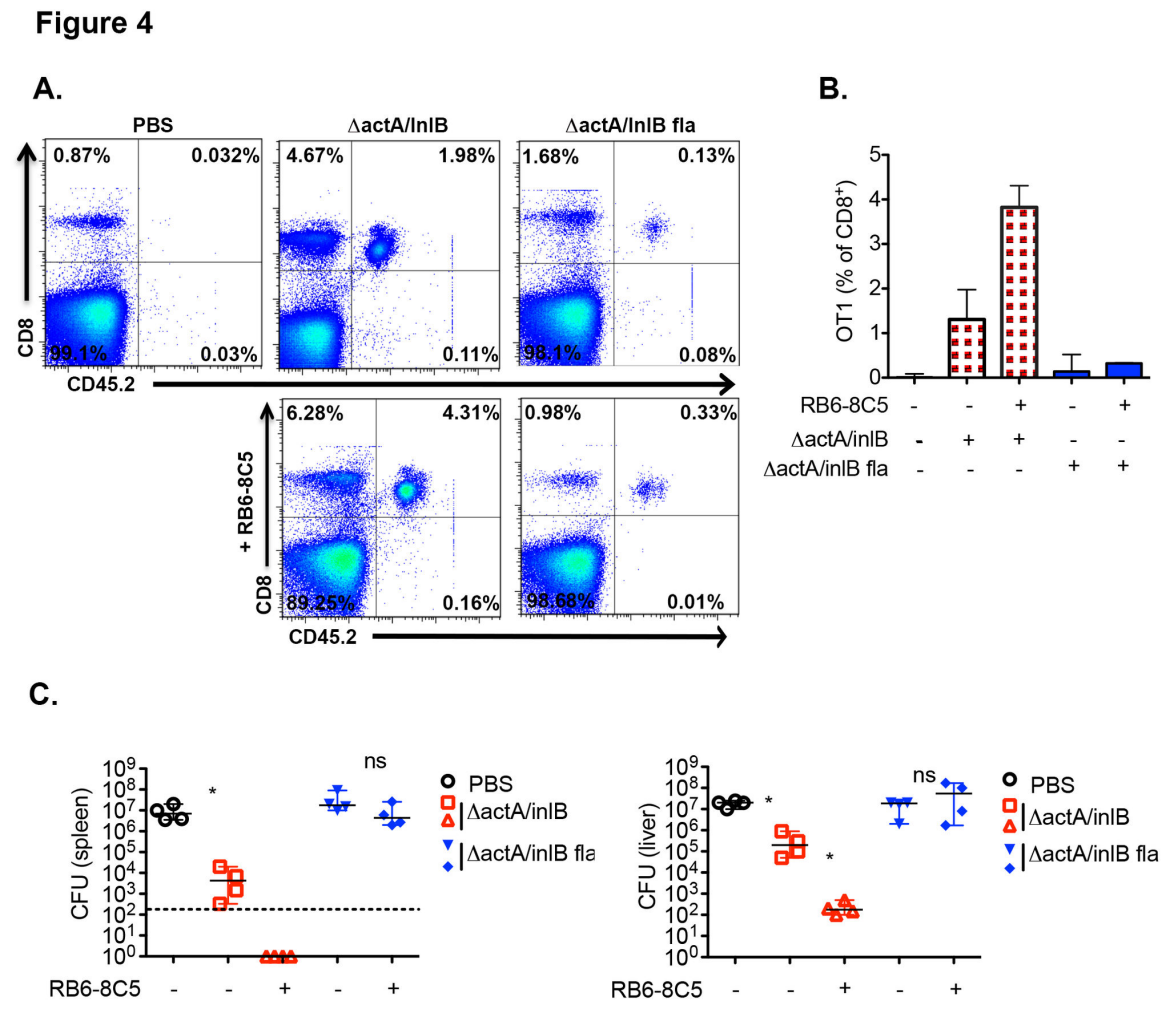
Inhibition of cell-mediated immunity despite MMC depletion. (A) 5x10^4^ naïve OT-I cells from C57BL/6 mice were transferred into 8-10 week old B6.SJL mice 24 hours prior to RB6-8C5 mAb (250µg) treatment. 5 hours after antibody treatment mice were infected intravenously with 1x10^4^ *ΔactA/InlB* or *ΔactA/InlB* L.p.FlaA *L. monocytogenes* both expressing ova peptide. OT-I percentages as analyzed by FACS from blood day 7 post-infection. Data representative of one experiment. (B) Bar graph (median plus range) of OT-I percentages as analyzed by FACS from blood day 7 post-infection. Data combined from three independent experiments. (C) 8-10 week old C57Bl/6 mice were treated with 250µg RB6-8C5 mAb and then immunized 5 hours later with 1x10^3^ *ΔactA/InlB* or *ΔactA/InlB* L.p.FlaA *L. monocytogenes*. 30 days post immunization mice were infected with 2x10^5^ wt *L. monocytogenes* for 3 days. Bacterial CFUs in liver and spleen day 3 post-infection. Dotted line – limit of detection. Data are representative of four independent experiments. *P < 0.05 by Mann-Whitney test.

Upon infection, CD8α^+^ DCs migrate into the white pulp and present antigen to CD8^+^ T cells up to 2 days post-infection [33]. During this time, neutrophils have also been seen to enter into the WP, make brief contacts with adjuvant matured DCs, and influence the level of antigen present in DCs as well as the length of time DCs interact with T cells during activation [29]. To analyze antigen-specific T cell activation, we adoptively transferred 5x10^4^ naïve OT-I CD8^+^ T cells from C57BL/6 into B6.SJL mice 24 hours prior to infection with *L. monocytogenes ΔactA/ΔinlB* Ova or *L. monocytogenes ΔactA/ΔinlB* L.p.FlaA Ova with and without RB6-8C5 mAb pre-treatment. The *ΔactA/ΔinlB* background was used to in order to control and normalize the increased rate of *L. monocytogenes* L.p.FlaA clearance. *L. monocytogenes ΔactA/ΔinlB* and *L. monocytogenes ΔactA/ΔinlB* L.p.FlaA have identical LD50s and are cleared at similar rates [23]. OT-I T cell expansion was monitored by determining the percentage of OT-I cells in the blood by FACS seven days post-infection. MMC depletion did not increase CD8^+^ T cell priming in *ΔactA/ΔinlB* L.p.FlaA *L. monocytogenes*-infected mice We compared the number of OT-I T cells based on the % of total mononuclear (Figure 4A,B). cells rather than the % of CD8 T cells due to the variability in the non-OT-I CD8 population (Figure 4A,B), which may be attributed to variable T cell apoptosis [38,39]. The starting OT-I population was 0.032±0.047%. *ΔactA/ΔinlB L. monocytogenes* infection yielded an OT-I population of 1.5±0.43% in the absence of MMC depletion and increased to 3.7±0.74% when MMC were depleted, as expected. *ΔactA/ΔinlB* L.p.FlaA *L. monocytogenes* infection yielded an OT-I population of 0.26±0.22% in the absence of MMC depletion and, surprisingly, only a slight increase to 0.30±0.038% when MMC were depleted. This was despite a significant increase in bacterial burden at 48 hours (Figure 3C) or 7 days (Figure S2) post *ΔactA/ΔinlB* L.p.FlaA *L. monocytogenes* infection with MMC depletion. As protection from secondary infection is a hallmark of the response to *L. monocytogenes*, we wanted to test whether MMC depletion would impact protection after a primary infection with *L. monocytogenes L*.p.*FlaA*.

To further test whether MMCs played a role in the inhibition of CD8^+^ T cell responses following inflammasome activation, we treated mice with RB6-8C5 antibody 5 hours prior to immunization with 1x10^3^ *L. monocytogenes ΔactA/ΔinlB* and *L. monocytogenes ΔactA/ΔinlB L*.p.*FlaA*. Mice were then challenged with 2x10^5^ (2xLD_50_) wt *L. monocytogenes* 30 days post immunization and 72 hours later bacterial colony counts were measured in the spleen and liver. In contrast to the enhanced protection generated in *L. monocytogenes ΔactA/ΔinlB* infected mice after MMC depletion, there was no effect of MMC depletion on the development of protective immunity following infection with inflammasome-activating *L. monocytogenes* (Figure 4C). These data suggest that although MMCs do regulate CD8^+^ T cell priming and recall responses following immunization, they do not play a role in inflammasome-mediated inhibition of antigen specific CD8^+^ T cell responses against *L. monocytogenes*. 

### Inflammasome activation reduces accumulation of *L. monocytogenes* in CD8α^+^ DC

**Figure 5:**
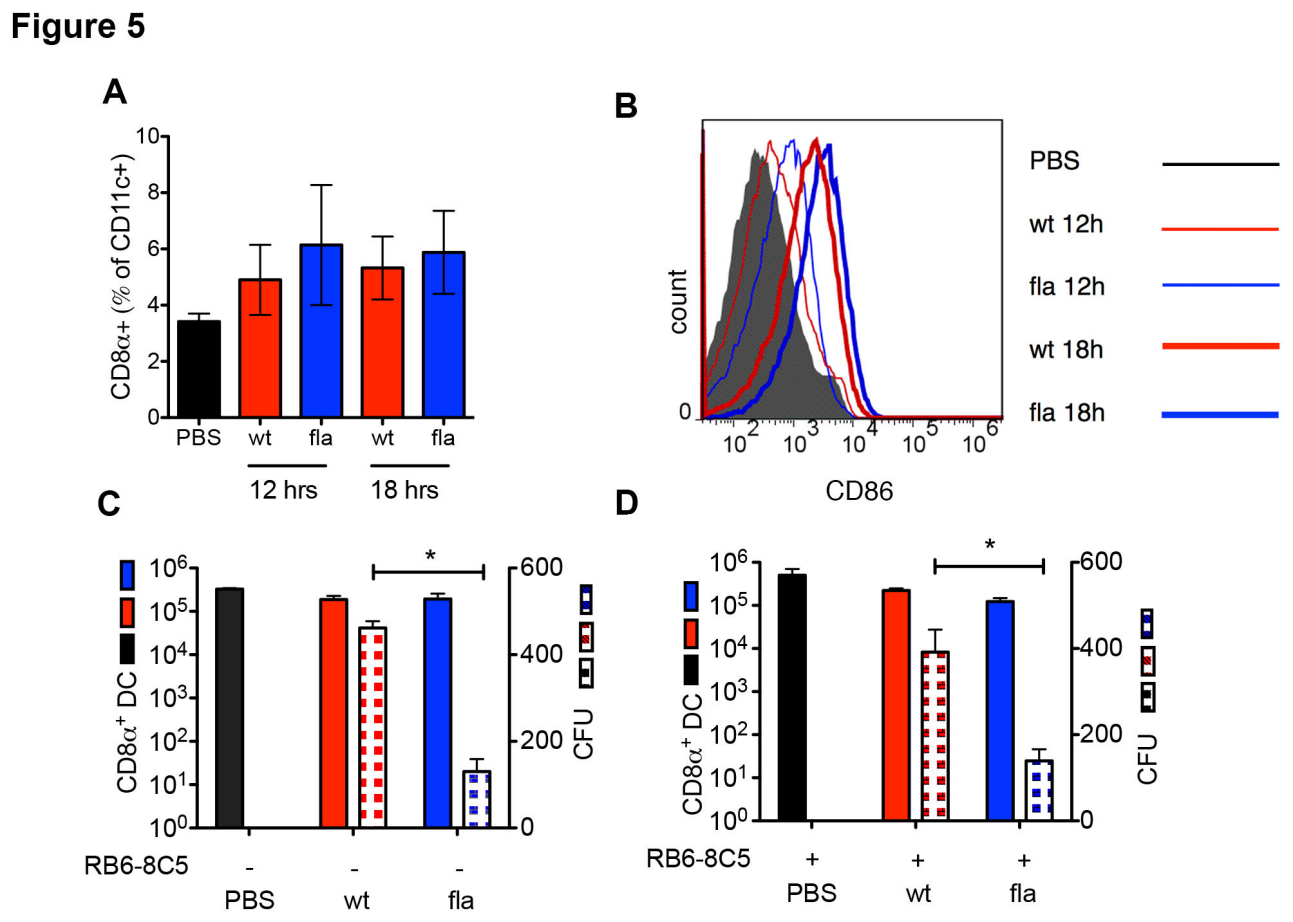
CD8α^+^ DCs harbor significantly less after *L. monocytogenes* inflammasome activation. (A) 8-10 week old C57Bl/6 mice were infected intravenously with PBS, 2.5x10^4^ wt or 1x10^5^ L. *monocytogenes* L.p.FlaA for 12 and 18 hours. After infection, spleens were harvested, dissociated, and isolated DCs were stained with CD8α^+^, CD11b^+^, CD11c+, CD86^+^ antibodies and analyzed using FACS. Bar graph of percentage of CD11c^hi^CD8α^hi^ DCs. The average ± SD of three replicates from 3 independent experiments. (B) Histogram of CD86 expression in CD11c^hi^CD8α^hi^ DCs. (C) 8-10 week old C57Bl/6 mice were infected intravenously with PBS, 2.5x10^4^ wt or 1x10^5^ L. *monocytogenes* L.p.FlaA for 24 hours. After infection, spleens were harvested, dissociated, and CD8α^+^ DCs were purified using bead purification. Total isolated spleens were plated on BHI with bacterial counts taken after 24 hours. The average ± SD of four replicates from 4 independent experiments. (D) C57Bl/6 mice were treated for 5 hours with 250µg RB6-8C5 mAb and then infected intravenously with PBS, 2.5x10^4^ wt or 1x10^5^ L. *monocytogenes* L.p.FlaA for 24 hours. After infection, spleens were harvested, dissociated, and CD8α^+^ DCs were purified using bead purification. Total isolated spleens were plated on BHI with bacterial counts taken after 24 hours. The average ± SD of four replicates from 4 independent experiments.

Previous studies found no reduction of DCs in spleens of mice infected with *L. monocytogenes* L.p.FlaA [23]. As a subset of conventional dendritic cells (DCs), CD8α^+^ DCs are efficient at cross presentation and carry *L. monocytogenes* from the MZ, the interface between splenic red and white pulp, to the T cell zones of the spleen [40,41]. The CD8α^+^ DCs are also known to be a reservoir for *L. monocytogenes* growth, so we focused on testing the impact of inflammasome activation on accumulation of *L. monocytogenes* in the CD8α^+^ DC subset. We infected B6 mice with 2.5 x 10^4^ *L. monocytogenes* or 1 x 10^5^ *L. monocytogenes* L.p.FlaA. Flow cytometry analysis of splenic DCs revealed that the CD8α^+^ DC were slightly increased in number by infection with no significant difference between *L. monocytogenes* and *L. monocytogenes* L.p.FlaA (Figure 5A). The CD8α^+^ DC were slightly more activated in the *L. monocytogenes* L.p.FlaA infected mice compared to *L. monocytogenes* based on CD86 expression (Figure 5B). After bead purification, bacterial colony counts were determined from isolated CD8α^+^ DCs. While CD8α^+^ DCs numbers were unchanged in uninfected mice compared to infected mice at 24 hours, we found a significant ~3-fold decrease in the amount of *L. monocytogenes* present in CD8α^+^ DCs in mice infected with *L. monocytogenes* L.p.FlaA compared to *L. monocytogenes* (Figure 5C). The significant decrease in CD8α^+^ DC bacterial burden may account for the poor immunogenicity of *L. monocytogenes L*.p.*FlaA*. Further, the depletion of MMCs prior to infection did not significantly increase the bacterial burden of CD8α^+^ DCs in mice infected with *L. monocytogenes* or *L. monocytogenes* L.p.FlaA (Figure 5D). These results emphasize the importance of the CD8α^+^ DC subset in priming of CD8^+^ T cell responses to *L. monocytogenes* and demonstrate that the bacterial burden in this critical subset is independent of the overall bacterial burden in the spleen or liver.

## Discussion

While inflammasome activation is critical for innate immune detection and defense against microbial pathogens [42-44], the changes in adaptive immunity that result from inflammasome activation remain unclear. Previously, we had shown that inflammasome activation inhibited the development of a robust cell-mediated immune response to *L. monocytogenes* [23]. Because of the role that MMCs play in bacterial containment and their recently described attenuation of T cell activation by contacts made with DCs [18-20], we hypothesized that MMCs played a role in the virulence defect seen in *L. monocytogenes L*.p.*FlaA*. We first established that the kinetics of MMC recruitment was altered following inflammasome activation. Next, we wanted to determine if MMCs could control the growth of inflammasome-activating *L. monocytogenes* and increase CD8^+^ T cell priming and protective immunity in mice infected with *L. monocytogenes L*.p.*FlaA*. While MMC depletion significantly increased *L. monocytogenes* L.p.FlaA titers in the liver and spleen, this recovery was only to the levels obtained with non-flagellin- expressing *L. monocytogenes* in the absence of MMC depletion. Even with the increased *L. monocytogenes* L.p.FlaA burden, a significant change was not seen in *L. monocytogenes* specific CD8^+^ T cell expansion on day 7 or protection on day 30 post-infection. Our data suggest that this defect may be the result of a significant decline in the bacterial burden of CD8α^+^ DCs that is left unchanged after MMC depletion. Our results suggest that while MMCs are recruited into the WP early and work to control *L. monocytogenes* L.p.FlaA in the T cell zones, they are not responsible for the virulence defect seen in *L. monocytogenes* L.p.FlaA or the inhibition of cell-mediated immunity. 

Previous results suggested that depletion of neutrophils had no effect on *L. monocytogenes* L.p.FlaA persistence in the liver and spleen [23]. We found that depletion of neutrophils and inflammatory monocytes led to a 2-3 log increase in CFU for both wt and L.p.FlaA *L. monocytogenes*. While appearing contradictory, this observation is actually consistent with the previous data that simply showed that there was no difference in the ratio of wt to L.p.FlaA *L. monocytogenes* following depletion. Our data more clearly show that while depletion of neutrophils and inflammatory monocytes allows for increased virulence of strains that activate the inflammasome, these cells alone are not responsible for the defect in virulence relative to wt *L. monocytogenes*. The partial effectiveness of MMCs in their function to control *L. monocytogenes* and the alterations in their infiltration into the splenic WP after inflammasome activation implies that triggering the inflammasome may induce changes in the microenvironment that supersedes the established role of MMCs in bacterial containment and clearance. Recent reports show that activation of Nlrc4 results in an “eicosanoid storm” from activated macrophages [45]. Eicosanoids, which are crucial for the activation of inflammation, could have a positive effect on host defense by increasing local vascular permeability at the site of infection, allowing for the influx of immune cells, such as MMCs that can kill bacteria [45,46]. Specifically, the release of leukotriene B_4_ (LTB_4_), a member of the eicosanoid family, has been shown to stimulate neutrophil chemotaxis, enhance neutrophil-endothelial interactions, and recruit neutrophils to the site of infection leading to the degranulation and release of superoxides [47-50]. Our results indicate that inflammasome activation alters the kinetics of MMCs when compared to mice infected with wt *L. monocytogenes*. It is possible that the release of eicosanoids, such as LTB_4_, are responsible for this alteration. 

Edelson et al. reports CD8α^+^ DCs as an obligate entry point for *L. monocytogenes* transport into splenic WP, with mice deficient in these cells showing a significant decline in bacterial burden in the spleen [41]. CD8α^+^ DCs showed a significant decline in bacterial burden after infection with *L. monocytogenes* L.p.FlaA despite slight increases in maturation state and numbers. There are a number of steps at which inflammasome activation could interfere with accumulation of bacteria in CD8α^+^ DC. The L.p.FlaA is expressed under control of actA regulatory elements such that it is unlikely that uptake of *L. monocytogenes* L.p.FlaA at the MZ is impaired. It is possible that induction of pyroptosis in CD8α^+^ DC that initially interact with *L. monocytogenes* L.p.FlaA in the MZ may release the live bacteria and force them to interact with new host cells. If these cells are less potent at priming CD8^+^ T cells, then this could account for the defects observed. It is also possible that any influence that inflammasome activation has on the CD8α^+^ DC population is not direct and may result from paracrine influence of other inflammasome altered cells types. MMCs were an initial candidate based on previous reports of their inhibition of antigen presentation through interaction with DCs in splenic WP as well as their inherent ability to compete for antigen [29,30]. Although the kinetics of MMC recruitment was altered by inflammasome activation, our results suggest MMCs are not required for the inhibition of T cell expansion and failure to form memory cells following immunization with *L. monocytogenes* L.p.FlaA. Other cell types whose function or frequency may be altered following inflammasome activation include NK cells and tissue macrophages. For example, COX-dependent prostaglandin E_2_ (PGE_2_) enhances the production of IL-10 by DCs, which down-regulates their function [51]. This model predicts that COX inhibitors may paradoxically enhance T cell responses to pathogens that induce robust inflammasome activation in DCs. 

While MMC depletion did not restore immunogenicity of *L. monocytogenes* L.p.FlaA we found that MMC depletion resulted in a increase CTL generation and protective immunity in response to ActA-deficient *L. monocytogenes* immunization. This indicates that MMCs normally attenuate T cell responses to *L. monocytogenes* as previously demonstrated [28-30]. Our results may also indicate that the role MMCs play in T cell activation is independent of the antigen level of CD8α^+^ DCs in infections with wt *L. monocytogenes*. Results from Yang et al. show impairment of DC-T cell interactions as a result of neutrophil competition for adjuvant proteins. Here we show that within the context of infection, MMC depletion has no significant influence over the number of viable *L. monocytogenes* in CD8α^+^ DCs during peak T cell priming, suggesting that contacts made by MMCs with DCs may disrupt DC-T cell interactions, possibly altering the level of antigen presentation or a reduction in key antigen presenting molecules [29]. This neutrophil-DC interaction is SIGN-R1 mediated and may also effect DC-T cell interaction time as well as the quality of the interaction [52].

We have shown that inflammasome activation accelerates MMC recruitment into splenic WP, but eventually extinguishes recruitment by 48 hours. Despite the role of MMCs in the containment of *L. monocytogenes* L.p.FlaA, MMCs are not the main mechanism by which CD8 T cells response to *L. monocytogenes* L.p.FlaA is attenuated. How inflammasome activation results in altered cell-mediated immunity remains unanswered. It is likely that inflammasome-mediated alterations in antigen level or presentation in CD8α^+^ DCs are responsible for these changes, as CD8α^+^ DCs show significant decrease in bacterial burden in *L. monocytogenes* L.p.FlaA-infected mice. Perhaps the release of eicosanoids by innate immune cells could lead to a influx of non-MMC innate immune cells in varying proportions that either compete with CD8α^+^ DCs for antigen or induce changes in CD8α^+^ DCs in a paracrine fashion. While our studies shed light on how inflammasome activation alters cell-mediated immunity, further research must be done to determine the mechanism by which Nlrc4 activation leads to alterations in CD8α^+^ DC accumulation of *L. monocytogenes* L.p.FlaA. Determining the yet unknown consequences of inflammasome activation will afford us a greater understanding of its role in the development of cell-mediated immunity, leading to a better design of vaccines that optimize inflammasome activation to promote robust cell-mediated immunity. 

## Methods

### Ethics Statement

This study was carried out in strict accordance with the recommendations in the Guide for the Care and Use of Laboratory Animals of the Public Health Service (National Institutes of Health). The New York University School of Medicine Institutional Animal Care and Use Committee (IACUC) approved this protocol. All surgery was performed under anesthesia. 

### Bacterial Strains and Infections

All *L. monocytogenes* strains used in this study were in the 10403s background, referred to as *L. monocytogenes* wild type (wt). Other *L. monocytogenes* strains are indicated by specific gene deletion (Δ) or addition. All *L. monocytogenes* strains were cultured in Brain Heart Infused media (BHI, Fisher). *L. monocytogenes* strain aliquots were kept at −80°C and grown in BHI for 3–4 hours until ~0.1 optical density (OD) at 600 nm. *L. monocytogenes* challenge doses were optimized for each strain, as reported in the text.

### Mouse Strains

LysM-EGFP, a gift of Dr. T. Graf and CD11c-EYFP mice, a gift of Dr. M. Nussenzweig, all on a C57BL/6 background were maintained in a colony in the specific pathogen-free Skirball Institute of Biomolecular Medicine at NYU Langone Medical Center (New York, NY). LysM-EGFP homozygous mice were bred with CD11c-EYFP homozygous mice to generate LysM-EGFP/ CD11c-EYFP mice. *L. monocytogenes* infected mice were housed under animal BSL2 conditions in a special room of the Skirball Institute specific pathogen-free facility. The NYU Langone Medical Center Institutional Animal Care and Use Committee approved all procedures.

### Myelomonocytic Depletion

For MMC depletion mice were treated with 250 μg of RB6-8C5 antibody (eBioscience) by intraperitoneal (i.p.) injection 5-6 hours prior to infection with *L.monocytogenes*.

### *Ex Vivo* Spleen Imaging

Mice were injected with 5 μg of 705 nm Quantum Dots (Invitrogen) or 4 μg dextran-tetramethlyrhodmine (TMR) (Invitrogen) 30 minutes prior to imaging in order to visualize the marginal zone (MZ). Explanted spleens were cut longitudinally in half using a vibratome (Ted Pella, Inc). Spleen sections were placed in a FCS2 chamber system (Bioptechs) used in an open configuration with a 1mm diagonal gasket. The FCS2 was connected to a pump and sections were perfused with 37°C RPMI-1640 media without phenol red bubbled with a mixture of 95% O_2_ and 5% CO_2_. Two photon images were acquired using a Zeiss 710 microscope with a 10X/0.45 objective. The 705 nm (quantum dots) and 540/566 nm (dextran) fluorescent signal was used to trace the MZ and define the WP cross-section for analysis of GFP intensity inside the WP as a relative measure of MMC recruitment. 

### CD8α^+^ dendritic cell maturation state and numbers assay

*L. monocytogenes*-infected spleens were excised, injected with 500 μl (1mg/ml) Collagenase D solution (Roche Diagnostics) and incubated for 45 minutes at 37°C. Spleen material was passed through 70 μm cell strainer to obtain a single cell suspension. Cells were labeled with CD8α^+^-PerCP-Cy5.5, CD11b^+^-eFluor405, CD11c^+^-APC, CD86^+^- Alexa 488 antibodies (eBioscience) and analyzed by FACs on a LSRII (BD Bioscience). FlowJo software (Tree Star) was used for data analysis. 

### CD8α^+^ dendritic cell bacterial titers

*L. monocytogenes*-infected spleens were excised, injected with 500 μL (1mg/ml) Collagenase D solution (Roche Diagnostics) and incubated for 45 minutes at 37°C. Spleen material was passed through 70 μm cell strainer to obtain a single cell suspension. CD8α^+^ DCs were purified from single cells suspension using a MACs CD8^+^ Dendritic Cell Isolation Kit (Miltenyi Biotec). Purified CD8α^+^ DCs were places in 0.05% Triton-X 100 for DC cell lysis and plated on Brain heart infused (BHI) agar plates to obtain *L. monocytogenes* colony counts. 

### T cell analysis

For the analysis of primary CD8^+^ T cells, naïve OT-I cells from C57BL/6 mice were transferred into B6.SJL mice 24 hours prior to intravenous infection with 1x10^4^ *ΔactA/inlB* or *ΔactA/inlB* L.p.FlaA *L. monocytogenes* (both expressing full length OVA epitope). At day 7 post-infection, OT-1 expansion was measured from total blood. Prior to labeling, red blood cells were removed using red blood cell lysing buffer. Cells were labeled with CD45.2 – APC and CD8 – FITC (eBioscience). FACs was performed on LSRII (BD Bioscience) and FlowJo software (Tree Star) was used for data analysis. 

### Protection Assays

8-10 week old male C57BL/6 mice were first treated with 250 μg of RB6-8C5 antibody for MMC depletion for 5 hours prior to infection with *L. monocytogenes* by i.p. injection. Mice were then immunized with 1x10^3^ of *ΔactA/inlB* or *ΔactA/inlB L. monocytogenes* L.p.FlaA (both expressing full length OVA epitope). Thirty days post immunization mice were challenged with 2x10^5^ *L. monocytogenes*. 72 hours post challenge, liver and spleens were harvested with 0.05% Triton-X 100 and plated in serial dilutions on brain-heart- infused agar plates to obtain colony counts. 

### Fluorescence Analysis and Statistics

Mean fluorescence was measured using Volocity software (Improvision). Mann-Whitney test was used to compare data from each group. Statistical analysis and graphing were done in Prism (GraphPad Software). Asterisk indicates *P*-value less than 0.05. 

## Supporting Information

Figure S1 **L.** ***monocytogenes* L.p.FlaA in WP after MMC depletion.**
8-10 week old CD11c-YFP transgenic x LysM^+/eGFP^ mice were treated with 250µg RB6-8C5 mAb and then infected i.v. with 2.5x10^4^ wt or 1x10^5^ L. *monocytogenes* L.p.FlaA both expressing RFP for up to 48 hours. After infection spleens were excised, sectioned, and image using multi-photon microscopy. (A) Gray-scale images of CD11c-YFP transgenic x LysM^+/eGFP^ mice at 48 hours after infection. *L. monocytogenes*-RFP, MMCs- blue, MZ-705 quantum dots-yellow.
(TIF)

Figure S2 **Day 7 post-infection bacterial titers after RB6-8C5 treatment.** 8-10 week old B6.SJL mice were treated with 250µg RB6-8C5 mAb for 5 hours prior to infection with 1x10^4^ *ΔactA/InlB* or *ΔactA/InlB* L.p.FlaA *L. monocytogenes*. At day 7 post-infection bacterial CFUs were collected from the spleen and liver. Dotted line – limit of detection. Data are representative of four independent experiments. *P < 0.05 by Mann-Whitney test.
(TIF)
