# Supplementary figures and images for: Inflammasome-Mediated Inhibition of Listeria monocytogenes-Stimulated Immunity Is Independent of Myelomonocytic Function
Source: PLoS One. 2013 Dec 9;8(12):e83191. doi: 10.1371/journal.pone.0083191 (PMC3857309; doi:10.1371/journal.pone.0083191)

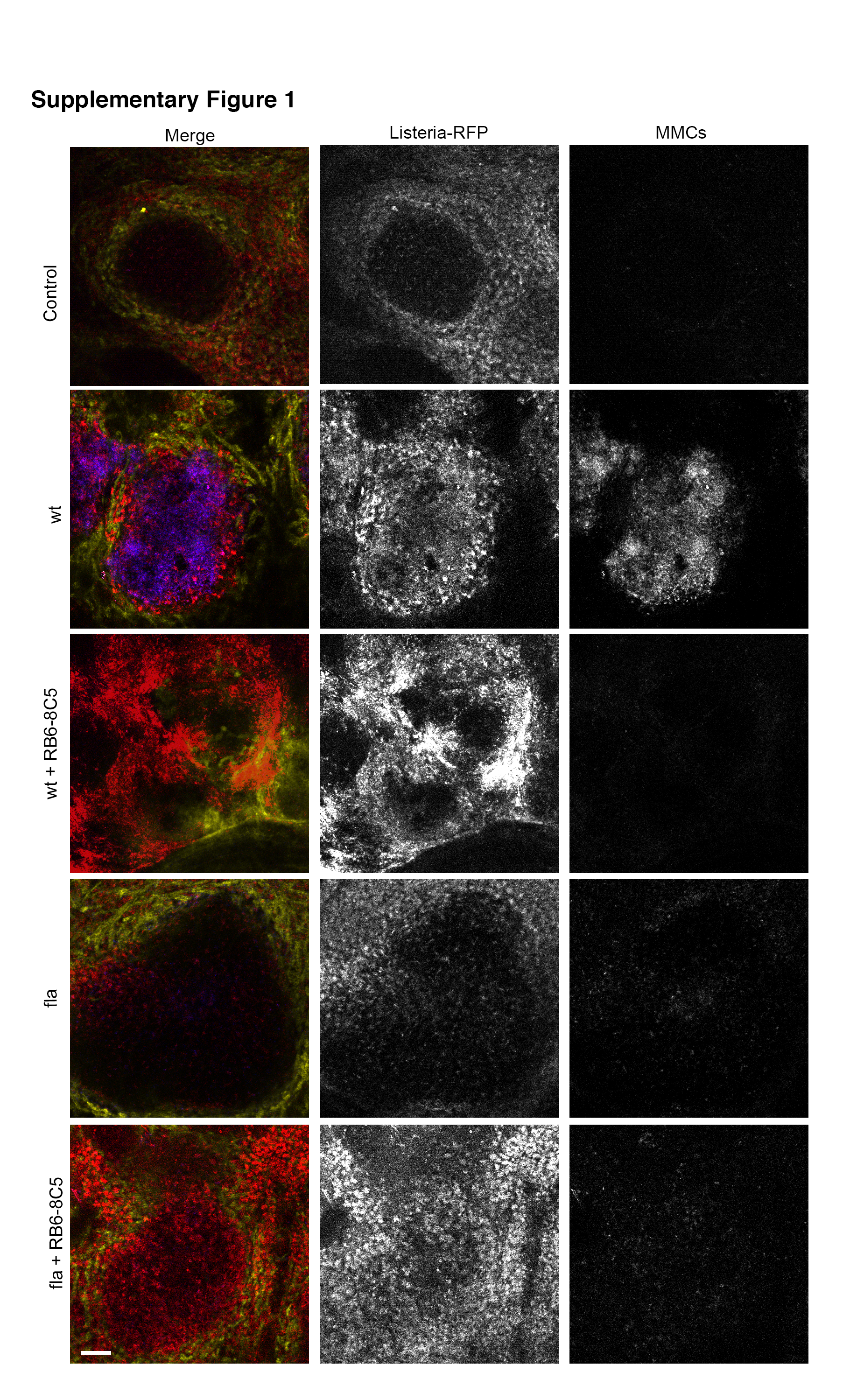

Supplement: Figure S1 — L. monocytogenes L.p.FlaA in WP after MMC depletion. 8-10 week old CD11c-YFP transgenic x LysM+/eGFP mice were treated with 250µg RB6-8C5 mAb and then infected i.v. with 2.5x10^4^ wt or 1x10^5^ L. monocytogenes L.p.FlaA both expressing RFP for up to 48 hours. After infection spleens were excised, sectioned, and image using multi-photon microscopy. (A) Gray-scale images of CD11c-YFP transgenic x LysM+/eGFP mice at 48 hours after infection. L. monocytogenes-RFP, MMCs- blue, MZ-705 quantum dots-yellow. (TIF) [file pone.0083191.s001.tif]

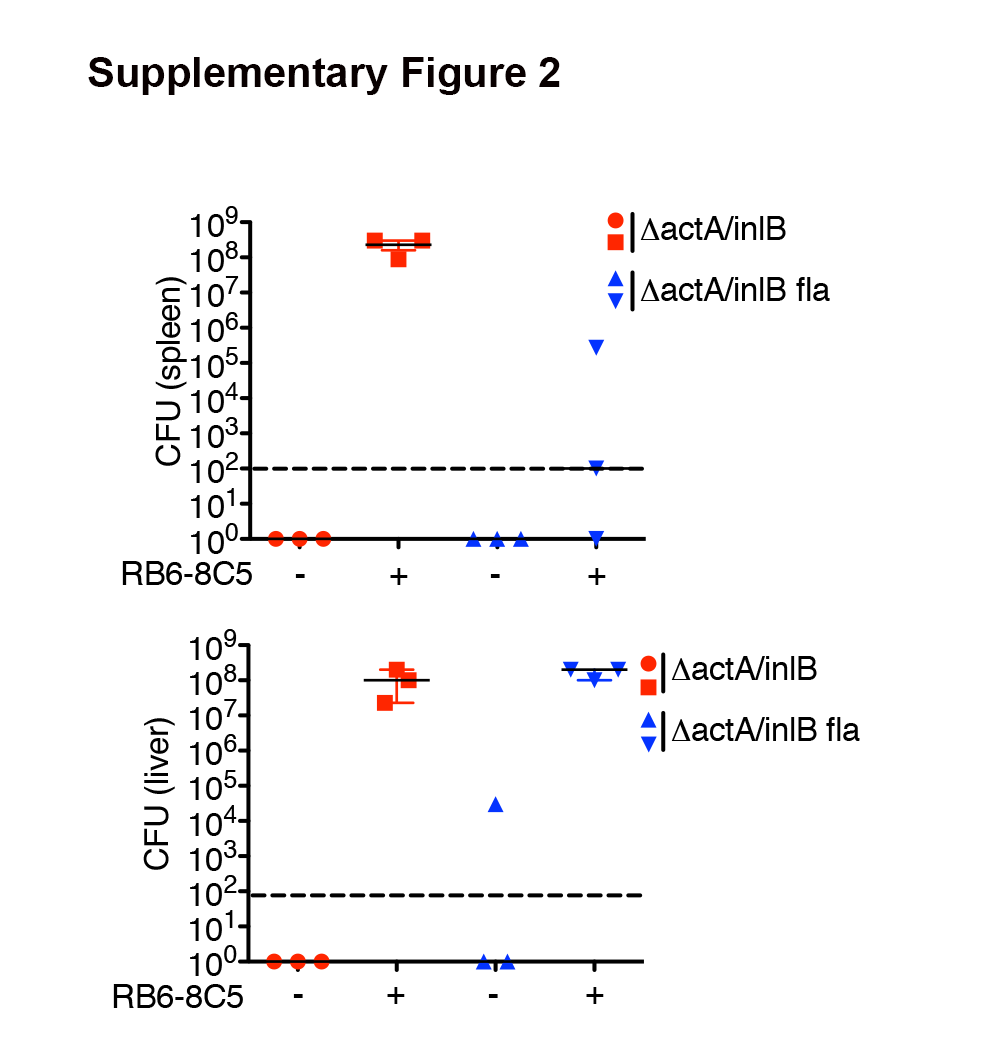

Supplement: Figure S2 — Day 7 post-infection bacterial titers after RB6-8C5 treatment. 8-10 week old B6.SJL mice were treated with 250µg RB6-8C5 mAb for 5 hours prior to infection with 1x10^4^ ΔactA/InlB or ΔactA/InlB L.p.FlaA L. monocytogenes. At day 7 post-infection bacterial CFUs were collected from the spleen and liver. Dotted line – limit of detection. Data are representative of four independent experiments. *P < 0.05 by Mann-Whitney test. (TIF) [file pone.0083191.s002.tif]
